# Towards a Vaccine against Plasmodium vivax Malaria

**DOI:** 10.1371/journal.pmed.0040350

**Published:** 2007-12-18

**Authors:** James G Beeson, Brendan S Crabb

## Abstract

The authors discuss a new study that suggests that*Plasmodium vivax* Duffy-binding protein could be a candidate antigen for developing a*P. vivax* vaccine.

There is little doubt that effective interventions against Plasmodium vivax are needed. An estimated 2.6 billion people live in areas endemic for P. vivax [1], and P. vivax carries a substantial burden of disease with 50–70 million clinical episodes each year [2].

## A Blood-Stage Vaccine for P. vivax?

Effective malaria vaccines could act by preventing initial liver-stage infection and/or blood-stage replication of parasites. The attraction of targeting blood stages is that it is during these stages that disease occurs. Although Plasmodium parasites initially infect hepatocytes, this pre-erythrocytic stage of malaria infection passes silently without any illness, and disease results from unrestricted replication of parasites in the blood. Vaccines targeting the blood stage may not have to induce sterile immunity (immunity that prevents infection per se), which is challenging to achieve; effective control of parasite replication may be sufficient to prevent illness and complications, as observed with naturally acquired immunity.

Despite decades of research and early promise, there are still no effective malaria vaccines available. There are many reasons why development of malaria vaccines has been so difficult. The multiple life cycle stages and estimated 5,000 genes of the parasite present a myriad of potential antigens, and it has been challenging to determine which of these antigens would make good vaccine targets. Additionally, antigens are highly polymorphic and expression and correct folding of recombinant proteins can be difficult to achieve.

## Duffy-Binding Protein as a Rational Vaccine Target

One antigen that stands out as an excellent vaccine candidate for P. vivax is Duffy-binding protein (DBP). In a new study published in this issue of *PLoS Medicine,* Brian Grimberg and colleagues provide important data to support further research on this antigen as a possible vaccine candidate [3].

**Linked Research Article:**

This Perspective discusses the following new study published in *PLoS Medicine:*

Grimberg BT, Udomsangpetch R, Xainli J, McHenry A, Panichakul T, et al. (2007) Plasmodium vivax invasion of human erythrocytes inhibited by antibodies directed against the duffy binding protein. PLoS Med 4(12): e337. doi:10.1371/journal.pmed.0040337

Christopher King and colleagues found that both rabbit and human antibodies inhibited binding of rPvDBPII to the Duffy antigen N-terminal region and to Duffy-positive human erythrocytes, suggesting that a PvDBP-based vaccine may reduce blood stage Plasmodium vivax infection.

DBP is a member of a family of related proteins found across different malaria species that are involved in host-cell invasion and adhesion. In P. vivax and the closely related simian malaria P. knowlesi, DBP binds to Duffy antigen/receptor for chemokines (DARC) during parasite invasion of reticulocytes [4,5]. DARC is a blood group antigen, expressed on the surface of reticulocytes and erythrocytes, and can also function as a receptor for several chemokines. P. vivax DBP (PvDBP) appears to be essential for invasion, and resistance to P. vivax is conferred by a lack of DARC expression [6,7], a phenotype common throughout African populations.

In the invasive merozoite form of the parasite, DBP is localized in apical secretory organelles known as micronemes [4], and is released just prior to the requirement for binding to DARC (Figure 1). This interaction is associated with the formation of a tight junction that moves over the surface of the merozoite as the parasite enters the erythrocyte. The solving of the DBP structure [8], combined with mutational analysis to identify key receptor-binding residues [9,10], has provided a sound understanding of the ligand–receptor interaction.

**Figure 1:**
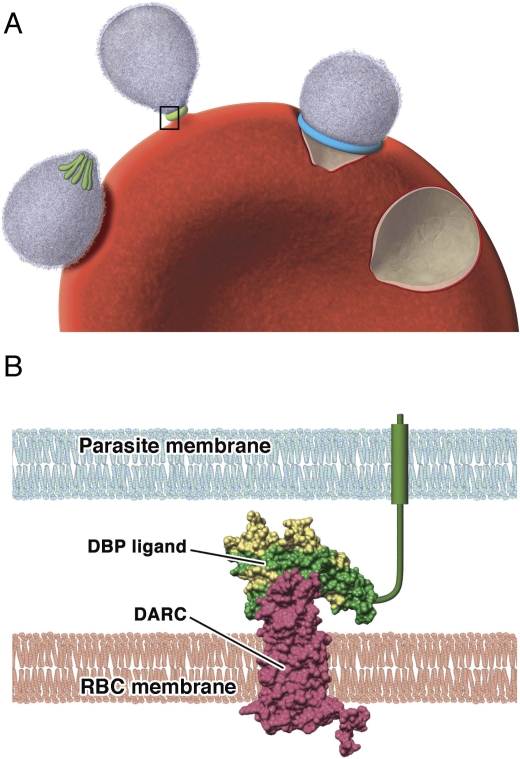
The role of Duffy-Binding Protein in the Invasion of Reticulocytes by P. vivax Merozoites (A) Step-wise invasion of reticulocytes involves initial attachment of the merozoite to the cell surface, resulting in deformation of the reticulocyte cell membrane; at this stage DBP is located in the intracellular micronemes (colored green) at the apical end of the merozoite. Reorientation of the merozoite then occurs such that the apical end is in contact with the reticulocyte membrane, and DBP is released for the formation of a tight junction. The tight junction (blue) then moves from the apical to posterior pole as the merozoite invades the cell, propelled by an actin–myosin motor. The reticulocyte membrane is finally re-sealed once invasion is complete. Invasion from initial attachment through to complete entry takes approximately one minute. (B) A model of the binding of PvDBP to the DARC receptor that occurs during invasion of reticulocytes by merozoites (inset from A). Polymorphic residues are colored yellow; conserved residues are colored green. Antibodies are predicted to bind to a polymorphic region of the DBP that is separate to, but may overlap with, the DARC-binding site. RBC, red blood cell (reticulocyte)

Most notably, the receptor-binding face of the protein has little polymorphism, whereas a region on an opposing face has many polymorphic residues, suggesting it is under selective pressure and is a target of acquired antibodies [8] (Figure 1). Hence it may be possible to develop a vaccine that predominantly targets the conserved region to block the PvDBP–DARC interaction.

## Understanding Antibody Function

Antibodies to merozoite antigens of malaria are thought to play an important role in acquired immunity, and may act by inhibiting erythrocyte or reticulocyte invasion and subsequent replication of parasites. However, data on the inhibitory activity of antibodies are limited, particularly for P. vivax.

Simply measuring antibodies to recombinant merozoite proteins in standard immunoassays has significant limitations, as recombinant antigens may not be in the same conformation or context as native protein. Standard immunoassays do not account for epitope specificity and antibody affinity, which are likely to be essential for inhibitory activity. A barrier to the development and use of functional assays for P. vivax is the difficulty of culturing the parasite in vitro. P. vivax does not efficiently invade mature erythrocytes, but instead is largely restricted to invasion of reticulocytes. This property limits its capacity to cause severe malaria, but also makes it very difficult to work with in the laboratory, which has held back progress on understanding P. vivax biology and vaccine development. However, recent approaches have been developed using cord blood or erythroid stem cells as a source of reticulocytes [11,12].

Grimberg and colleagues now show that antibodies to recombinant PvDBP generated by vaccination of rabbits or affinity-purified from human serum can effectively inhibit reticulocyte invasion by P. vivax [3]. The same antibodies that inhibit invasion also inhibit binding of recombinant PvDBP to DARC. This suggests that the invasion-inhibitory activity of the antibodies is mediated by blocking the PvDBP–DARC interaction, and not by interfering with potential interactions that may occur between other molecules and PvDBP, or the PvDBP–DARC complex.

Invasion inhibition by antibodies to PvDBP may seem somewhat surprising since, based on the structure of PvDBP, antibodies are predicted to target a region that is separate from the DARC binding site [8]. Given the extremely rapid process of invasion (about one minute [13]), and the fact that PvDBP is probably not released and exposed until attachment to reticulocytes, antibodies against PvDBP may have limited opportunity to exert a receptor-blocking effect. However, until the molecular details of PvDBP–DARC binding are better understood, it is difficult to confidently predict the significance of polymorphisms or the mechanism of antibody function. It is possible that the DARC and antibody binding sites have substantial overlap sufficient for antibodies to inhibit binding [14].

These findings provide an important proof-of-concept that anti-PvDBP antibodies can inhibit merozoite invasion. Further studies are needed to: (1) understand the acquisition and induction of inhibitory antibodies; (2) relate the concentration of antibodies required for effective inhibition to antibody concentrations in vivo; and (3) determine the relationship between inhibitory antibodies and antibody reactivity in standard immunoassays using recombinant antigen. It would be valuable to learn whether antibodies that block recombinant PvDBP–DARC binding predict protection in human studies or animal challenge models.

## Moving Forward to Vaccine Trials

Should a vaccine targeted to PvDBP now go forward to a phase I trial? Although PvDBP is a rational vaccine choice, there remain important issues to address. An effective vaccine may need to overcome significant antigenic diversity in PvDBP. Grimberg and colleagues report that anti-PvDBP antibodies bound at similar levels to two different PvDBP variants. However, it will be important to extend this work to evaluate the effect of polymorphism on antibody inhibition. Others reported that some polymorphisms did affect the activity of antibodies that inhibit binding of PvDBP to DARC [15].

Another challenge is finding and evaluating suitable adjuvants that induce appropriate antibody responses and are safe for use in humans. Many animal vaccine studies are performed using powerful adjuvants that are not registered for use in humans. For example, a PvDBP vaccine in monkeys was partially effective with Freund's adjuvant, which cannot be used in humans, but not with Montanide ISA720, which is licensed for human studies. Generating functional antibodies is an important vaccine objective and it would seem logical that the induction of invasion-inhibitory antibodies should be part of the criteria for a successful phase I PvDBP vaccine trial to proceed to phase II. Testing antibodies for inhibition of PvDBP–DARC binding may prove to be a suitable alternative for the more challenging live-cell invasion inhibition assay.

## Abbreviations

DARC: Duffy antigen/receptor for chemokines
DBP: Duffy-binding protein
PvDBP: Plasmodium vivax Duffy binding protein
